# DexA70, the Truncated Form of a Self-Produced Dextranase, Effectively Disrupts *Streptococcus mutans* Biofilm

**DOI:** 10.3389/fmicb.2021.737458

**Published:** 2021-09-28

**Authors:** Nan Liu, Xin Li, Maofeng Wang, Fengyu Zhang, Chuandong Wang, Kundi Zhang, Hongwei Wang, Sujuan Xu, Wei Hu, Lichuan Gu

**Affiliations:** State Key Laboratory of Microbial Technology, Shandong University, Qingdao, China

**Keywords:** dental caries, *Streptococcus mutans*, biofilm, polysaccharides, DexA70, lysozyme

## Abstract

Billions of people suffer from dental caries every year in spite of the effort to reduce the prevalence over the past few decades. *Streptococcus mutans* is the leading member of a specific group of cariogenic bacteria that cause dental caries. *S. mutans* forms biofilm, which is highly resistant to harsh environment, host immunity, and antimicrobial treatments. In this study, we found that *S. mutans* biofilm is highly resistant to both antimicrobial agents and lysozyme. DexA70, the truncated form of DexA (amino acids 100–732), a dextranase in *S. mutans*, prevents *S. mutans* biofilm formation and disassembles existing biofilms within minutes at nanomolar concentrations when supplied exogenously. DexA70 treatment markedly enhances biofilm sensitivity to antimicrobial agents and lysozyme, indicating its great potential in combating biofilm-related dental caries.

## Introduction

Dental caries is the most common biofilm-mediated disease of the oral cavity caused by some cariogenic bacteria and remains the most prevalent chronic disease in both children and adults (Petersen et al., 2005; Karhumaa et al., 2021). From the data of the American National Health and Nutrition Examination Survey in 2018, 59% of adolescents 12--19 and 92% of adults 20 to 64 have suffered from dental caries in their permanent teeth (NIDCR^1^).

*Streptococcus mutans* is the major causative agent of dental caries and the leading member of a specific group of cariogenic bacteria that form a biofilm on the surface of teeth (Hamada and Slade, 1980; Loesche, 1986). Formation of biofilm is a key virulence feature of *S. mutans*. The bacteria in the biofilm produce acids, which demineralize the dental surface (Venault et al., 2014), promoting the formation of dental plaque and periodontitis (Loe et al., 1965; Socransky, 1979; Dahlen, 1993; Marsh, 1994; Nishihara and Koseki, 2004).

Biofilms are highly organized and structured communities of bacterial cells enmeshed in an extracellular matrix of variable density and composition (Costerton et al., 1999; Branda et al., 2005; Valm, 2019). In nature, most of the biofilms develop from initial microbial attachment on a surface followed by formation of cell clusters and further development and stabilization of the micro-colonies, which are occurring in a complex extracellular matrix (Watnick and Kolter, 2000; Branda et al., 2005). The majority of biofilms matrix is polysaccharides, and dental biofilms are no exception; up to 40% of the dry weight of dental biofilm is composed of polysaccharides (Paes Leme et al., 2006).

*Streptococcus mutans* utilizes dietary sucrose to synthesize extracellular polysaccharides (EPS) to promote biofilm formation (Koo et al., 2010; Bowen et al., 2018). The EPS matrix, consisting of an α-(1–6)-linked glucose polymer with α-(1–3) branch linkages, plays a crucial role in regulating the formation and virulence of cariogenic biofilm by influencing the physical and biochemical properties of biofilm (Schilling and Bowen, 1992; Flemming et al., 2016). It may promote accumulation and adherence of microorganisms and accelerate coherence of bacterial cells to each other and to apatitic surface, and thereby modulates the initial steps of cariogenic biofilm development and proliferation and facilitates the formation of mature dental plaque (Long and Edwards, 1972; Tanzer, 1985; Rolla, 1989; Marsh, 2003; Koo et al., 2009). In addition, to be a reserve source of energy, EPS protects microorganisms from inimical influences, affects diffusion of substances into and out of biofilm, and helps to concentrate metal ions and other physiological nutrients within a microenvironment (Tatevossian, 1990; Wilson and Ashley, 1990; Hayacibara et al., 2004; Leme et al., 2006; Flemming and Wingender, 2010). As a physical barrier, it hinders the diffusion of antibiotics and host-driven antimicrobial factors into the deepest layers of biofilm, enhancing the resistance of microorganisms in biofilm (Costerton et al., 1981; Brown and Gilbert, 1993; Shen et al., 2016).

Current approaches to reduce caries include mechanical cleaning, sealants, fluoride applications, and antibacterial agents with a broad-spectrum antiseptic action like chlorhexidine (Mandel, 1988; Yousefimanesh et al., 2015). Although effective in the short term, these therapies all bring side effects. It was reported that the use of fluoride had a potential to cause major adverse human health problems, while having only a modest dental caries prevention effect (Peckham and Awofeso, 2014). Chlorhexidine is often present in different formulations such as mouthwashes, gels, galenic preparations, or dentifrices (Haseeb et al., 2016; Zhou et al., 2016). However, the role of chlorhexidine in preventing dental caries is controversial, because it can cause stains on the teeth, particularly on the resin of the fillings on caries (Oteri et al., 2011; Gostemeyer et al., 2017). Moreover, the use of antiseptics has been shown to perturb the commensal microbiome, increasing pathogenic characteristics and cytotoxicity to host cells and causing other detrimental effects on oral health (Chatzigiannidou et al., 2020).

In recent years, increasing emphasis has been placed on more preventive and human-friendly caries treatments with minimal side effects such as the use of probiotics and prebiotics (Guarner et al., 2005; Caglar et al., 2006), or natural substances like herbal lollipop containing licorice extracts (He et al., 2006; Hu et al., 2011; Chen et al., 2019; Aires et al., 2020). Other studies have attempted to remove the major causative agent of the caries, *S. mutans*, from the biofilm community, known as a specifically targeted antimicrobial peptide (Eckert et al., 2006; He et al., 2010; Kaplan et al., 2011), but there are also limitations existing in their therapeutic utility. The data still disagree as regards their performance in non-physiological conditions (da Silva et al., 2012; Jorge et al., 2012).

The application of enzymes, such as lysozyme, has been considered as an alternative strategy to fight various kinds of biofilms. However, the effects are far from satisfactory (Hukic et al., 2018). It has been reported that lysozyme used at the concentrations of tens of micrograms per milliliter prevented biofilm formation but had little effect on resolving the existing biomass (Stuchell and Mandel, 1983; Wang and Germaine, 1991; Gottschick et al., 2016).

Even though these treatments have been applied and have reduced the prevalence of caries over the past few decades, the consequence of dental caries continues to grow. The presence of biofilm produced by some cariogenic bacteria, especially *S. mutans*, is the major barrier for antibacterial agents to function effectively (Bowden and Hamilton, 1998; Mah et al., 2003; Hathroubi et al., 2017). The best treatment must be able to eradicate biofilms (Jakubovics et al., 2021).

Therefore, polysaccharides, especially (1–3) and (1–6)-α-D-glucans, as the key structural and functional constituents of the *S. mutans* biofilm matrix, have become appealing antibiofilm targets for novel therapeutic strategies. A variety of glucanohydrolases, such as mutanase and dextranase, from fungi and bacteria have become important active component of oral hygiene products such as mouthwashes, toothpastes, and chewing gum to support mechanical cleaning of teeth (Caldwell et al., 1971; Wiater et al., 2008). Theoretically, dextranase can inhibit biofilm formation by hydrolyzing α-1,6-glucosidic bonds within water-soluble polysaccharides. However, the presence of enzymes only had a modest effect on total amount of glucan formed (Otsuka et al., 2015). This result implies that the polysaccharides in biofilm may be arranged in a way quite different from the purified form. To achieve maximum activity, these enzymes must be able to penetrate into biofilm and get access to the cleavage site easily.

In our previous work, we found that PslG, a self-produced protein in *Pseudomonas aeruginosa*, prevents biofilm formation and efficiently disassembles existing biofilms when supplied exogenously at nanomolar concentrations (Yu et al., 2015; Pestrak et al., 2019). The success of PslG reminded us that the self-produced enzymes may be the best candidate for treatment since it is produced to function in biofilms. Luckily, *S. mutans* also produces a dextranase, DexA, which hydrolyzes α-1,6-linkages of dextran and produces isomaltooligosaccharides (IGs) of various sizes. DexA is crucial in the process of biofilm formation and thought to be responsible for the ecology of dental plaque: hydrolyzing the glucans as potential storage polysaccharides to supply nutrients for bacteria metabolism and controlling the amount and content of extracellular glucans to make it more indurative and adhesive in nature (Igarashi et al., 2002). We supposed DexA might be potentially useful for dental caries prevention. Nevertheless, Otsuka et al. (2015) reported that the effect of DexA was limited. We therefore sought to identify enzymes that selectively target and degrade EPS. In this study, we find that the truncated DexA (DexA70, 100–732) shows a much higher activity in inhibiting biofilm formation and disrupting a pre-formed biofilm when supplied exogenously. Even more striking, DexA70 treatment sensitizes biofilm bacteria to lysozyme and other antibacterial agents, leading to the improved eradication of *S. mutans* biofilm. In addition, based on our results, DexA70 could be developed as an effective treatment with less side effects for dental plague in the future.

## Materials and Methods

### Strains and Cultures

*Streptococcus mutans* UA159 was obtained from the Institute of Microbiology, Chinese Academy of Sciences and grown in brain–heart infusion broth (BHI) at 37°C under 5% CO_2_ (v/v). Solid media were prepared by adding 1.5% (w/v) agar. For biofilm formation, *S. mutans* cells were grown in BHI supplemented with 1% sucrose (w/v) in 96-well plates under static conditions.

### Cloning, Protein Expression, and Purification

Sequences encoding full-length DexA (Signal peptide is not included) and DexA70 (residues 100–732) were amplified and cloned into pGL01, a vector modified from pET15b by introducing a PreScission Protease (PPase) cleavage site for the removal of His-tag. The resulting expression plasmids were transformed into *Escherichia coli* BL21 (DE3), which was cultured at 37°C until OD600 reached 0.8 and then induced overnight with 0.3 mM isopropyl β-D-thiogalactopyranoside at 16°C.

For protein purification, bacterial cells were harvested by centrifugation for 15 min at 5000 × *g*. The bacterial cells were resuspended in lysis buffer (25 mM Tris-HCl, pH 8.0, 200 mM NaCl) and lysed by sonication. The lysate was centrifuged at 25,200 × *g* for 45 min to remove the cell debris. The supernatant was loaded onto a Ni-NTA column (GE Healthcare) for affinity chromatography. Miscellaneous proteins were removed by washing several times with the lysis buffer. The resin binding the target protein was mixed with PPase (with a final concentration of 0.1 mg/ml) overnight at 4°C to remove the His-tag. The protein sample was then eluted with lysis buffer. For further purification, protein was loaded onto an ion-exchange column (Source 15Q HR 16/10, GE Healthcare) and eluted with a linear gradient of 0–1 M NaCl in 25 mM Tris-HCl buffer pH 8.0. Finally, the protein sample was purified by size-exclusion chromatography (Superdex 200 10/300 GL, GE Healthcare) in 10 mM Tris-HCl buffer, pH 8.0, containing 100 mM NaCl. Protein purity was examined by SDS-PAGE.

### Verification of the Susceptibility of Planktonic Bacteria and Biofilm to Chlorhexidine and Cetylpyridinium Chloride

To determine the effects of chlorhexidine (CHX) and cetylpyridinium chloride (CPC) on planktonic bacteria, overnight cultures of *S. mutans* were diluted by 1:100, then inoculated into fresh BHI media containing different concentration of CHX or CPC, and grown at 37°C, 5% CO_2_. The cultures were sampled at 1-h interval, and the absorbance at the wavelength of 600 nm was tested.

The effects of antimicrobial agents on biofilms were tested by calculating remaining viable cells after treatment with CHX and CPC. Twelve-hour biofilm was washed by saline solution and then different concentrations of CHX or CPC were added and incubated for 30 min. After reaction, biofilm was scraped off the tubes and resuspended in saline solution. Serially diluted suspensions were plated onto BHI agar plates and anaerobically incubated for 48 h at 37°C. The amount of viable bacterial cells from different conditions was expressed as CFU/ml of biofilm. All experiments were performed in triplicate.

### Characterization of the Recombinant Dextranase

Dextranase activity was measured by monitoring the release of reducing sugar during incubation of the mixture containing 200 nM dextranase, 1% dextran 40,000, and 50 mM sodium phosphate buffer (pH 5.0) at 37°C. The reaction was sampled at 2-min interval for 30 min. The sampled reaction was terminated by adding 2 ml of 3,5-dinitrosalicylic acid (DNS). Controls were made by preparing a solution containing the same ingredients except that the enzyme was boiled in advance. Mixtures of both the samples and controls were boiled for 5 min and then deionized water was added to a volume of 10 ml. Absorbance was measured at a wavelength of 540 nm. The gradients in the linear region were calculated to measure enzyme activity.

To determine the optimum temperature for the recombinant dextranase, enzymatic activity was measured at 0, 10, 20, 30, 35, 40, 45, 50, 55, 60, 70, and 80°C, respectively. To study the influence of pH on dextranase activity, enzymatic activity was measured in different buffers at different pH: 50 mM acetic acid sodium acetate buffer (pH 3.5–5.0), 50 mM phosphate buffer (pH 5.0–7.0), and 50 mM Tris-HCl buffer (pH 7.0–9.0).

### Biofilm Inhibition or Disassembly Assay

The cultures were incubated statically in 96-well plates for 12 h at 37°C to allow biofilm formation. For the biofilm inhibition assay, enzymes were supplied into the growth medium at the beginning of inoculation. After incubation, non-adherent cells and matrix were removed by gently washing the plate with deionized water. The wells were stained with 100 μl of 0.1% (w/v) crystal violet for 10 min and then deionized water was added to rinse the wells three times to remove the unbound dye. The remaining dye was solubilized by addition of 200 μl of 30% (v/v) acetic acid and shaking for 15 min. The absorbance was measured at the wavelength of 595 nm. The amount of biofilm was proportional to the absorbance of the staining crystal violet.

For biofilm disassembly assays, 12-h-old biofilm was washed with distilled water to remove the non-adherent cells and medium. The wells were filled with 200 μl of 50 mM phosphate buffer (pH 5.0) with varying concentrations of DexA70 or lysozyme. Reactions were allowed to proceed for up to 30 min at 37°C, and then quenched by washing the plates with distilled water. The wells were stained with 100 μl of 0.1% (w/v) crystal violet for 10 min and then washed with distilled water. The crystal violet bound to biofilms was solubilized with 200 μl 30% acetic acid for quantification. Experiments were performed in triplicate separately.

### Quantification of Biofilm Biomass

To obtain the biomass from the biofilms, *S. mutans* UA159 biofilm was grown in sterile glass tubes in BHI broth at 37°C under 5% CO_2_ (v/v) for 12 h. Biofilm was then washed twice with saline solution to remove unattached cells and then treated with or without DexA70 and lysozyme in 50 mM PBS buffer (pH 5.0) at 37°C for 30 min. The treated biofilm was washed twice with saline solution to remove dispersed cells. The remaining biofilm was scraped off the tubes and resuspended in 1 ml of saline solution. The suspension was transferred to pre-weighted tubes and incubated with 100% ethanol at −20°C for 10 min and then centrifuged at 25,200 × *g* for 30 min at 4°C. The procedure was repeated once, and the precipitate was washed with 75% ethanol and dried for 24 h in a desiccator. Biomass value was calculated by subtracting the initial weight of the empty tube from the final dry weight and expressed as milligrams per milliliter of biofilm suspension.

### Image Acquisition and Analysis of Biofilms by Confocal Laser Scanning Microscope

For confocal laser scanning microscopy observation, biofilm was grown in a petri dish with glass bottom and treated with enzymes. After treatment, the buffer was gently removed and the biofilm was stained with polysaccharide stain concanavalin A (ConA) lectin conjugated with tetramethylrhodamine for visualization of dextran and DNA stain SYTO9 for all bacteria and propidium iodide (PI) for dead cells (Molecular Probes, Invitrogen). Z-stack images were acquired on a Confocal Laser Scanning Microscope (CLSM) (LSM900, Zeiss, Germany) and further analyzed by imaris software package for the creation of three-dimensional visualizations. For systematic analysis of biofilm architecture and structure, a computer program COMSTAT with a wide variety of functions for handling, analyzing, and displaying images was used to quantify the elements of biofilm including bio-volume, thickness distribution, mean thickness, and surface-to-volume ratio (Heydorn et al., 2000).

### Minimum Biofilm Inhibition Concentration and Minimum Biofilm Eradication Concentration Measurement

Minimum biofilm inhibition concentration (MBIC) and minimum biofilm eradication concentration (MBEC) of biofilm were determined based on Clinical and Laboratory Standard Institute (CLSI) (2018) guidelines. Measurement was performed using a modified version of the Calgary biofilm device method (Ceri et al., 1999; Reiter et al., 2013). Biofilm was formed through overnight incubation at 37°C. Non-adherent cells were removed by gentle washing three times with sterile saline solution. Biofilm was exposed to media or media containing 500 nM DexA70 for 30 min before challenge of serial antibiotics and then washed again with sterile saline solution. The treated biofilm was left to air dry for 15 min. Serial twofold dilutions of each antimicrobial agent in BHI broth were then added and incubated at 37°C for 24 h. MBIC represents the minimal antimicrobial concentration at which there was no observable bacterial growth in wells containing treated biofilm (OD600 < 0.1).

For MBIC measurement, after being washed, biofilm treated with or without DexA70 was exposed to the serial diluted antimicrobial agents for 1 h. The media were then removed and wells were washed three times with sterile saline solution. Finally, antimicrobial-free BHI was added, followed by incubation for 24 h at 37°C. MBEC was defined as the minimal antimicrobial concentration at which bacteria fail to regrow after antimicrobial exposure (OD600 < 0.1).

### Measurement of Minimum Biofilm Viable Cells Eradication Concentration

To accurately measure the effect of DexA70 on minimal concentrations of CHX and CPC to completely kill *S. mutans* in biofilm, biofilm was cultured in BHI broth with 1% sucrose for 12 h, and gently washed three times followed by antibiotics challenges with/without 500 nM DexA70 for 30 min. After treatment, biofilm was washed and scraped off the tubes and resuspended in 0.9% NaCl. Serially diluted suspensions were plated onto BHI agar plates in triplicate and anaerobically incubated for 48 h. Bacteria growth was measured by colony-forming units (CFU). Minimum biofilm viable cells eradication concentration (MBVEC) was defined as minimal antimicrobial concentration at which no colony grows on plate.

## Results

### Biofilm Enhances Bacterial Resistance to Antibacterial Agents

**FIGURE 1:**
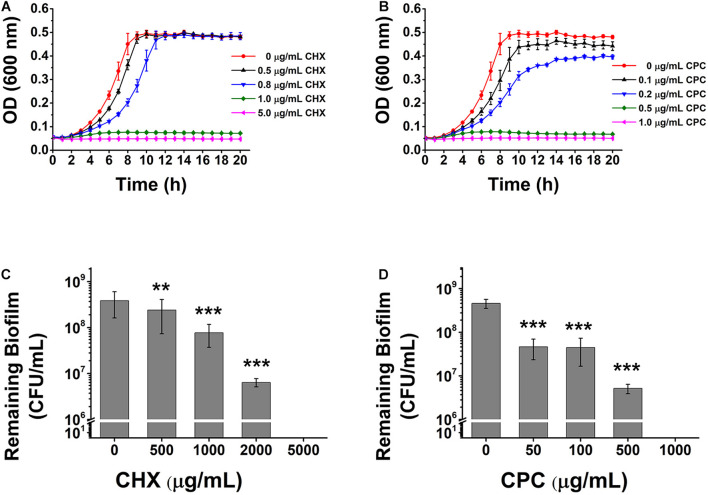
The effects of CHX and CPC on *S. mutans* planktonic cells or biofilm. The planktonic cell growth curve and viable cells remaining in biofilms after treatment were tested. **(A,B)** The growth curves of *S. mutans* in BHI media containing different concentrations of CHX **(A)** or CPC **(B)**. **(C,D)** CFU enumeration for remaining viable cells in biofilms treated with CHX **(C)** or CPC **(D)**. Means ± SD from at least three independent experiments with technical triplicates are shown. Student’s *t*-test by SPSS 15 was used for data analysis. A *p*-value < 0.05 was considered statistically significant. **p < 0.01; ***p < 0.001.

Chlorhexidine and cetylpyridinium chloride, agents commonly used in mouthwashes to treat dental caries, are known to be the most effective treatment currently applied in the dental field. It was reported that the concentrations of CHX and CPC contained in mouthwashes in the market (0.1 and 0.03% to 0.05%, respectively) are much higher than the sensitivity values of *S. mutans* (So Yeon and Si Young, 2019). To figure out if there is a possibility of reducing the antimicrobial agents in the mouthwashes, we tested the susceptibility of planktonic bacteria and biofilm to CHX and CPC respectively. 1 μg/ml (0.0001%) of CHX (Figure 1A) and 0.5 μg/ml (0.00005%) of CPC (Figure 1B) inhibit planktonic cells growth thoroughly, which are 1‰ of both agents used in mouthwashes. However, the effective concentrations to kill bacteria wrapped in biofilm is 0.5% for CHX, 5,000-fold higher than that against planktonic cells (Figure 1C) or 0.1% for CPC, 2,000-fold higher than that against planktonic cells (Figure 1D). This proves that biofilm formation dramatically promotes the bacterial drug resistance to antimicrobial agents.

### *Streptococcus mutans* Biofilm Is Fully Resistant to Lysozyme

**FIGURE 2:**
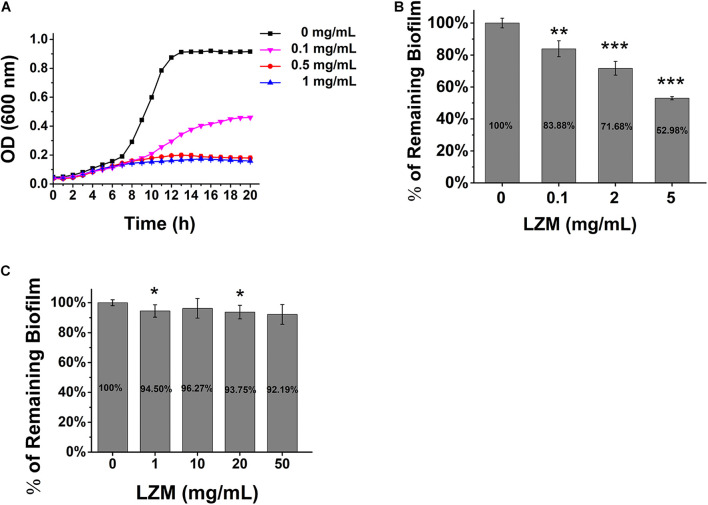
Lysozyme inhibits *S. mutans* growth and biofilm formation. **(A)** The growth of *S. mutans* in BHI broth in various concentrations of lysozyme. **(B)** The quantification of biofilm formation in various concentrations of lysozyme. **(C)** Pre-formed biofilm treated with various concentrations of lysozyme. Statistical significance is indicated as compared with untreated biofilms using a *t*-test. All of the values shown represent the mean ± standard deviation of the results from three independent experiments. *p < 0.05; **p < 0.01; ***p < 0.001.

Enzymatic targeting of biofilms by lysozyme has been considered as an alternative treatment for biofilm infection in certain bacteria, which generally have no side effect caused by antiseptics. The effect of lysozyme on *S. mutans* growth and biofilm formation was tested. The results showed that lysozyme had significant antimicrobial activity against the planktonic cells (Figure 2A) and hindered the biofilm formation at higher concentrations (Figure 2B), but it was not able to degrade the already formed biofilm (Figure 2C).

### DexA70 Has the Potential to Be an Effective Strategy to Treat Dental Caries

High resistance of *S. mutans* biofilm to antimicrobial agents and lysozyme imply that disrupting biofilm would be a better way to make an effective treatment. Since dextran is the major component of *S. mutans* biofilm EPS, dextranase and mutanase from various bacterial sources have been used to treat dental biofilms. However, most of them had little effect (Pleszczynska et al., 2010; Wiater et al., 2011; Wang et al., 2016).

The success of PlsG in *P. aeruginosa* biofilm degradation suggests that self-produced enzymes may be better candidates for combating the biofilm-related infections (Yu et al., 2015). Just like *P. aeruginosa*, *S. mutans* does encode a polysaccharide hydrolase, DexA, which plays an important role in biofilm formation. The functional similarity between PslG and DexA imply that DexA may also be a good treatment. DexA was then overexpressed and purified in *E. coli* BL21(DE3). The ability of DexA to hydrolyze *S. mutans* biofilm was tested. The results showed that although DexA degraded *S. mutans* biofilm to some extent, the effect was just not so good as expected (dispersed less than 50% of biofilm).

DexA belongs to glycoside hydrolase family 66, consisting of five regions from the N- to the C-termini: the N-terminal signal peptide sequence (N-terminal 24 amino acids), variable region (25–99), catalytic region (100–615), glucan-binding site (616–732), and C-terminal region (733–850). It has been reported that full-length DexA was easily digested into many peptide fragments by proteases, and the protein lacking of both N- and C-terminal regions showed higher catalytic activity (Kim et al., 2011). The crystal structure of this fragment (100–732, Dex70) has been determined, and the catalytic mechanism has also been elucidated (Suzuki et al., 2012). This reminds us that Dex70 may have better performance in *S. mutans* biofilm degradation.

### DexA70 Prevented Biofilm Formation While Supplied Exogenously

DexA70 was expressed and purified as previously mentioned (Suzuki et al., 2012). Hydrolysis activity was measured by using DNS colorimetric method under different pH values and different temperatures. The optimum pH of DexA70 is at pH 5.0 (Supplementary Figure 1A), and the optimum temperature of DexA70 is at 40°C (Supplementary Figure 1B).

**FIGURE 3:**
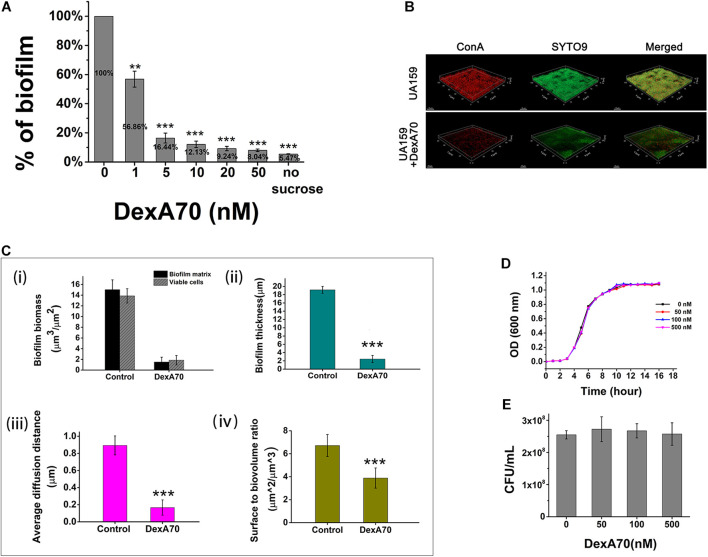
DexA70 inhibits biofilm formation without affecting bacterial growth. **(A)** DexA70 inhibited UA159 biofilm formation in a dose-dependent manner. Inhibition effect was calculated by the ratio of remaining biofilm biomass post DexA70 treatment to that of untreated control. **(B)** Exogenous DexA70 efficiently prevented the biofilm formation by UA159. Images were 1-day-old biofilm grown with/without DexA70 and corresponding CLSM z-stack images (dextran in red stained by ConA and viable cells in green stained by SYTO9). 63 × oil immersion magnification, scale bar = 15 μm. **(C)** Morphological parameters were evaluated by processing CLSM stack images of biofilms, cultivated with/without DexA70. (i). Biofilm biomass and viable cell biomass. (ii). biofilm thickness. (iii). Average diffusion distance of biofilm. (iv). Surface to biovolume ratio of biofilm. Data were calculated from three independent measurements and were reported as average. **(D,E)** The planktonic growth curve **(D)** and the colony-forming unit enumeration **(E)** of UA159 in BHI media with various concentrations of DexA70. The exogenous DexA70 does not affect cell growth. Statistical significance is indicated as compared with negative control using a *t*-test. **p < 0.01; ***p < 0.001.

To determine the biofilm-inhibiting activity of DexA70, DexA70 was added to the culture medium at the beginning of incubation. Biofilm biomass decreased significantly compared with the controls (Figure 3A). IC50 (the concentration that can inhibit 50% of biofilm biomass) of DexA70 was ∼1 nM. The biofilm biomass of UA159 treated with 50 nM DexA70 was similar to that of the negative controls, which had no sucrose as substrate, indicating that DexA70 at 50 nM completely prevents biofilm formation.

The effect of DexA70 was further quantitatively characterized by CSLM images. EPS was stained in red by concanavalin A conjugated with tetramethylrhodamine and viable cells were stained in green by SYTO9. Markedly, the pellicles with DexA70 treatment lost about 99% of dextran (Figure 3B). The topographic surfaces, biofilm organization, and architecture were then systematically analyzed by using the COMSTAT plugin of ImageJ (Figure 3C). The architectural analysis of DexA70-treated biofilm showed 90 ± 5.9% reduction of the biofilm biomass and 86.7 ± 6.3% reduction of the viable cells in the biofilm matrix as compared with the untreated biofilm (Figure 3Ci). Similar effects were also observed for the biofilm thickness (Figure 3Cii) and the average diffusion distance (Figure 3Ciii). The reduction of surface-to-volume ratio suggested that the presence of DexA70 inhibited biofilm formation and caused architecture disruption (Figure 3Civ). The biofilm inhibition effect of DexA70 is not due to the inhibition of the bacterial growth since the addition DexA70 did not change the growth rate of *S. mutans* (Figures 3D,E).

### Exogenous DexA70 Dispersed Existing Biofilms

**FIGURE 4:**
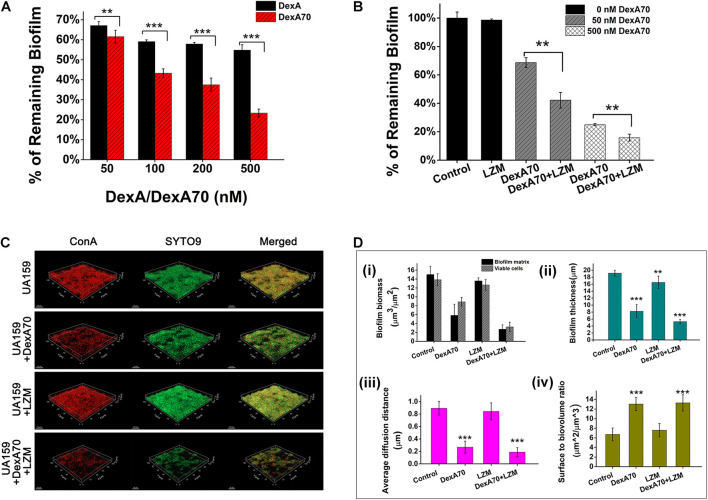
Synergistic effect of DexA70 and lysozyme (LZM) on biofilm treatment. **(A)** The comparison between DexA and DexA70 in biofilm dispersion. Enzymes were added at 50, 100, 200, and 500 nM; the activity of DexA70 is much higher than DexA. Means ± SD are shown. **(B)** DexA70 disassembled 12 h biofilm in a dose-dependent manner with or without 2 mg/ml lysozyme. Means ± SD are shown. **(C)** The three-dimensional CLSM images for 12 h *Streptococcus mutans* biofilm treated with/without DexA70 and lysozyme (stained in red by ConA and green by SYTO9). **(D)** Quantitative analysis of biofilm structure in different conditions containing the biomass, mean thickness, thickness distribution, and surface-to-volume ratio. (i). Biofilm biomass and viable cell biomass. (ii). biofilm thickness. (iii). Average diffusion distance of biofilm. (iv). Surface to biovolume ratio of biofilm. Data were calculated from three independent measurements and were reported as average. Student’s *t*-test was used to conduct statistical analyses, and differences were considered significant when *p < 0.05. **p < 0.01; ***p < 0.001.

Dextran is the major component of *S. mutans* biofilm and confers adherence and drug resistance onto the bacteria. Since DexA70 is able to prevent biofilm formation by degrading dextran, we think that it may also be able to disperse the existing biofilm. The activity of DexA70 on *S. mutans* biofilm was then tested. The results showed that the biofilm disassembly activity of DexA70 increased with concentration. DexA70 dispersed 43 ± 2.2% of biofilm at 50 nM and 77 ± 2% of biofilm at 500 nM in 30 min. By comparison, full-length DexA had a much lower activity at the same concentration (Figure 4A). Deletion of the N- and C-terminal regions may make the catalytic site more exposed and get access to the substrate easier, thus resulting in more potent activity on *S. mutans* biofilm.

### Co-administration of DexA70 and Lysozyme Showed a Synergistic Effect in Biofilm Disassembly

The measurement of biofilm formation ability and cell growth showed that lysozyme kills planktonic bacteria but not biofilm bacteria and has no degradation effect on preformed biofilm, while DexA70 disperses biofilm without affecting bacterial growth (Figures 2, 3). Thus, we speculate that co-administration of DexA70 and lysozyme should have a synergistic effect on biofilm. To test the synergistic effect of DexA70 and lysozyme,biofilm was cultured in BHI broth with 1% sucrose for 12 h, and treated with the following measures separately: 500 nM DexA70; 2 mg/ml lysozyme; 500 nM DexA70 plus 2 mg/ml lysozyme. After 30-min incubation at 37°C, the reaction buffers were removed and the treated biofilms were subjected to testing by three methods. The amount of remaining biofilm was quantitatively measured by staining with 0.1% crystal violet. The results showed that co-administration of DexA70 and lysozyme resulted in as low as 15.85 ± 2.4% remaining biofilm (Figure 4B). The degradation rate of the biofilm by co-administration of DexA70 and lysozyme was much higher than the sum effects of DexA70 and lysozyme.

To visualize the effect of the treatments, the biofilms formed in each condition were stained by fluorescent dyes and characterized by a CLSM, and further analyzed by COMSTAT. The three-dimensional biofilm images also confirmed the synergistic effect of DexA70 and lysozyme in biofilm disruption. The biofilm untreated with enzymes were thick, compact, and uniform, whereas co-administration of DexA70 and lysozyme resulted in porous biofilms containing channels and voids (Figure 4C). Quantitative measurements of biofilm morphology are indicated in Figure 4D. The treatment of enzymes caused 81.8 ± 6.3% reduction of biofilm biomass and 76.6 ± 4.4% reduction of viable cells. The biofilm thickness and average diffusion distance were also decreased. Most strikingly, the surface-to-volume ratio of biofilm matrix treated with DexA70 and lysozyme increased greatly. The surface-to-volume ratio is the surface area divided by the bio-volume which reflects what fraction of the biofilm is in fact exposed to the environment. The significant erosion of the surfaces created a great deal of microcavities, which largely increased the contact of biofilm with the environment, causing higher sensitivity to environmental changes and challenge of antibiotics.

### DexA70 and Lysozyme Makes a Dispersing and Killing Strategy

**FIGURE 5:**
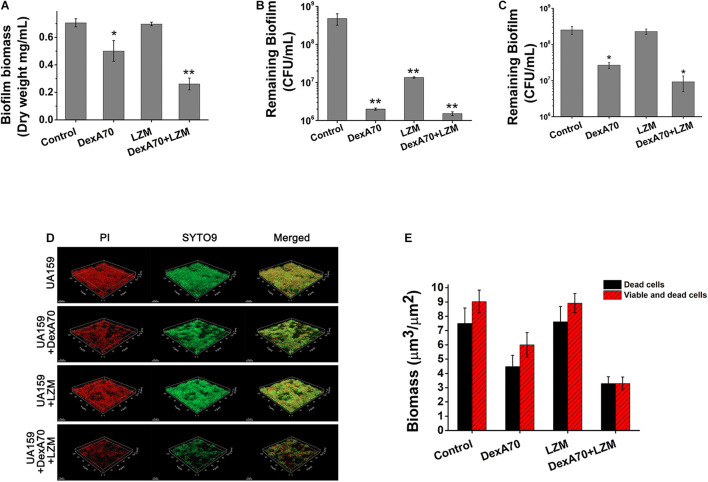
DexA70 and lysozyme make a dispersing and killing strategy. **(A)** The dry weight of UA159 biofilm treated with/without DexA70 and lysozyme. Co-administration of DexA70 and lysozyme efficiently reduced biofilm biomass. **(B,C)**. CFU enumeration for remaining viable cells in biofilms treated with/without DexA70 and lysozyme. **(B)** Adding DexA70 and lysozyme (LZM) at the beginning of biofilm formation. **(C)** Adding enzymes after biofilm formation and incubating for 30 min. Experiments were performed in triplicate, and the results were shown as the mean ± SD. **(D)** LIVE/DEAD BacLight bacterial viability assay. Images show 12 h biofilm and cells prior to (0 min) and post 30 min of DexA70 and lysozyme treatment. Live bacteria are stained in green by SYTO 9 and dead bacteria are stained in red by propidium iodide. 63 × oil immersion magnification, scale bar = 15 μm. **(E)** Biomass (μm^3^/μm^2^) of alive and damaged cells was calculated by processing CLSM stack images. Experiments were performed in triplicate, and the results are shown as the mean ± SD. Statistical significance is indicated as compared with negative groups untreated with enzymes using a *t*-test. *p < 0.05; **p < 0.01.

To make clear the synergistic mechanism of DexA70 and lysozyme, we measured the dry weights of biofilm and the number of viable cells of *S. mutans* in remaining biofilms after being treated in different ways. The biofilm treated with both DexA70 and lysozyme has the least remaining biomass (Figure 5A). To evaluate the viable cells, the remaining biofilm after treatment was washed with saline solution and then resuspended, serially diluted, and plated onto BHI agar plates in triplicate. The number of viable bacteria was counted by CFUs. As expected, both DexA70 and lysozyme treatments significantly reduced the total viable bacteria number in the remaining biofilms when added at the beginning of biofilm formation. Co-administration of DexA70 and lysozyme reduced the number of viable bacteria even further. In the case of existing biofilm, however, DexA70 was still effective while lysozyme completely lost killing activity. Strikingly, co-administration of DexA70 and lysozyme still showed a synergistic effect in reducing the number of viable bacteria in the remaining biofilm. This strongly suggests that co-administration of DexA70 and lysozyme makes a dispersing and killing strategy. Although *S. mutans* biofilm is fully resistant to lysozyme, hydrolysis of the glucan by DexA70 exposes the cells to lysozyme for killing (Figures 5B,C). This assay also eliminated the interference from planktonic cells, which provides a more accurate measurement for the effect of a specific treatment on biofilm.

To get a better understanding of the dispersing and killing mechanism, we carried out a LIVE/DEAD BacLight bacterial viability assay, in which all bacteria were stained in green by SYTO9 and dead bacteria were stained in red by PI. The untreated biofilm and biofilms treated by DexA70, lysozyme or both were examined on a CLSM (Figure 5D). A corresponding quantitative evaluation of live and dead cells from the morphological analysis of z-stacks is shown in Figure 5E. It is noteworthy that the synergistic effect of dispersing and killing is obvious since treatment with both enzymes resulted in a much more complete biofilm disruption; both the live and dead bacteria were mostly removed.

### DexA70 Treatment Sensitized *Streptococcus mutans* Biofilm to Antiplaque Agents

**FIGURE 6:**
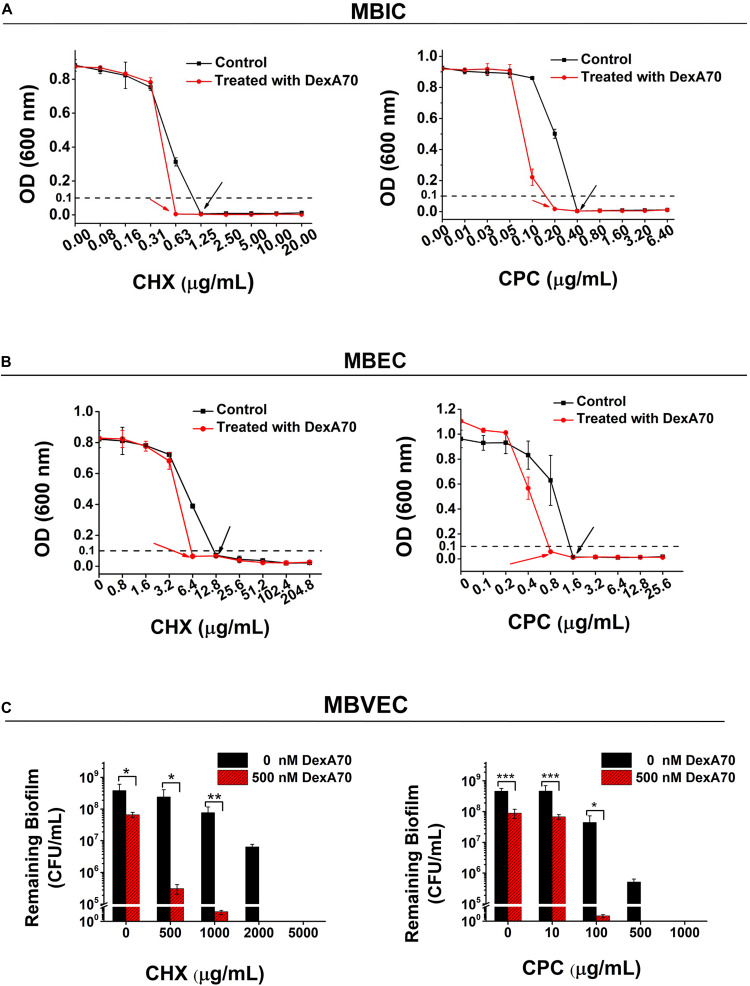
DexA treatment improved biofilm sensitivity to Chlorhexidine and Cetylpyridinium Chloride. MBIC **(A)** and MBEC **(B)** of biofilm treated with (⚫) and without (■) DexA. Arrows indicate the corresponding values of MBIC and MBEC. **(C)** MBVEC of biofilm treated with CHX (left) or CPC (right) and with/without DexA70. Means ± SD are shown. *t*-test was performed for testing differences between groups. *p < 0.05; **p < 0.01; ***p < 0.001.

The effectiveness of the dispersing and killing strategy implies that DexA70 treatment may also sensitize *S. mutans* biofilm to commonly used antiplaque agents. To verify this hypothesis, we tested the MBIC (minimum biofilm inhibition concentration) of CHX and CPC against DexA70-treated 12-h-old biofilm. For both antimicrobial agents, DexA70-treated biofilm has a twofold lower MBIC in comparison of untreated biofilm (Figure 6A). To know whether antiplaque agents are able to eradicate the remaining surface-attached biofilm after 30 min of DexA70 treatment, we examined the MBEC (minimum biofilm eradication concentration). Similarly, for both antimicrobial agents, DexA70-treated biofilm has a twofold lower MBEC (Figure 6B).

The data of MBIC and MBEC also suggest a dispersing and killing mechanism. However, due to the complexity and heterogeneity of biofilm, MBIC and MBEC cannot quantitatively and accurately reflect the effectiveness of DexA70 treatment. We think that the number of viable *S. mutans* cells in the biofilm with different treatment would better reflect the effectiveness of dispersing and killing strategy. The result showed that, for both CHX and CPC, the minimal concentrations to completely kill *S. mutans* in biofilm [minimum biofilm viable cells eradication concentration (MBVEC)] treated with DexA70 were fivefold lower than those of untreated groups (Figure 6C). This indicated that DexA70 treatment sensitized *S. mutans* biofilm to antiplaque agents and formed a killing and dispersing strategy.

## Discussion

Dental caries, as a *S. mutans* biofilm-related disease, has long been the most common health problem that affects people throughout their lifetime and poses a major health burden for many countries (Petersen et al., 2005; Karhumaa et al., 2021). In this study we find that *S. mutans* biofilm is highly resistant to antimicrobial chemical reagents. Even more serious *S. mutans* biofilm is also fully resistant to lysozyme. This implies that an effective treatment with minimal side effect needs to disrupt biofilm specifically and efficiently.

Since (1–3) and (1–6)-α-D-glucans are the major components of the *S. mutans* biofilm matrix, a variety of glucanohydrolases, such as mutanase and dextranase, have been used to treat dental caries as the active components of oral hygiene products (Caldwell et al., 1971; Wiater et al., 2008). However, the therapeutic effect is very limited. The success of PslG in the treatment of *P. aeruginosa* biofilm suggests that self-produced enzymes may have better activity.

DexA is an essential protein in the synthesis of EPS, a key biofilm matrix exopolysaccharide in *S. mutans*. DexA has also been used as a potential strategy in combating biofilms, but the effect was poor. In this study, we find that the truncated form of DexA (DexA70, 100–732) prevents the biofilm formation and efficiently disrupts a pre-formed biofilm when supplied exogenously. Degradation of the biofilm matrix not only disassembles bacterial community structure but also may expose the bacteria to lysozyme for killing and allowing antimicrobial reagents to diffuse into biofilm easier, thus making a dispersing and killing strategy. The bactericidal and biofilm degradation effects of co-administration of DexA70 and lysozyme were greater than when lysozyme or dextranase were tested alone (Figures 4B, 5C). DexA70 treatment greatly increases the susceptibility of the biofilm bacteria to CHX and CPC in comparison with the untreated biofilm (Figure 6).

The dispersing and killing strategy of DexA70 with lysozyme or chemicals has great advantages over other treatments. Firstly, the combination of DexA70 and lysozyme excludes the use of any antimicrobial chemical reagent, thus avoiding related side effects. Ingesting a little bit of enzyme by accident during treatment would not cause any negative effect since these two enzymes are proteins that will be digested in the stomach. Secondly, the combination of DexA70 and CHX or CPC brought more obvious bactericidal effect and would also reduce related side effects greatly since the use of chemical reagents are markedly decreased compared with common mouthwashes. Misuse and overuse of these antimicrobial chemical reagents might be harmful to gut microbiota, as 1,000-fold diluted mouthwash is sufficient to inhibit planktonic cells of the tested bacteria (Figures 1A,B).

Nowadays, MBIC and MBEC are common measures used for assessing effectiveness of biofilm treatment. However, once absorbed by the biofilm matrix, chemicals would not be washed off easily. This, in turn, affects the accuracy of MBIC and MBEC. In this study, we find that MBVEC, the minimum biofilm viable cells eradication concentration, is a better measure. Since biofilm will be disrupted completely and smeared onto plate, this minimizes the negative effect of the residual chemical reagents.

To be effective, sufficient interactions between the enzymes and dental plaques are necessary. Chewing gum might be the best choice as an effective local delivery system for continuously releasing the enzymes. A chewing gum containing DexA70 and lysozyme followed by mouthwash containing antimicrobial chemical reagents of lower concentration have great potential to be a better solution for dental caries with minimized side effects. Further studies of animal and clinical trials are necessary to demonstrate the safety and effectiveness of using enzymes in dental care.

## Data Availability Statement

The datasets presented in this study can be found in online repositories. The names of the repository/repositories and accession number(s) can be found in the article/Supplementary Material.

## Author Contributions

NL, WH, and LG designed the experiments and wrote the manuscript. NL conducted the all experiments and data analysis with the help of XL, MW, FZ, KZ, HW, CW, and SX. LG supervised the project. All authors commented on the manuscript.

## Conflict of Interest

The authors have filed a patent application on the use of DexA70.

## Publisher’s Note

All claims expressed in this article are solely those of the authors and do not necessarily represent those of their affiliated organizations, or those of the publisher, the editors and the reviewers. Any product that may be evaluated in this article, or claim that may be made by its manufacturer, is not guaranteed or endorsed by the publisher.

## Footnotes

1
http://www.nidcr.nih.gov/
