# Supplementary material for: DexA70, the Truncated Form of a Self-Produced Dextranase, Effectively Disrupts Streptococcus mutans Biofilm
Source: Front Microbiol. 2021 Sep 28;12:737458. doi: 10.3389/fmicb.2021.737458 (PMC8505985; doi:10.3389/fmicb.2021.737458)
Supplement: Supplementary file 1 [file Data_Sheet_1.docx]

**Supplementary Information**

**Figure S1**


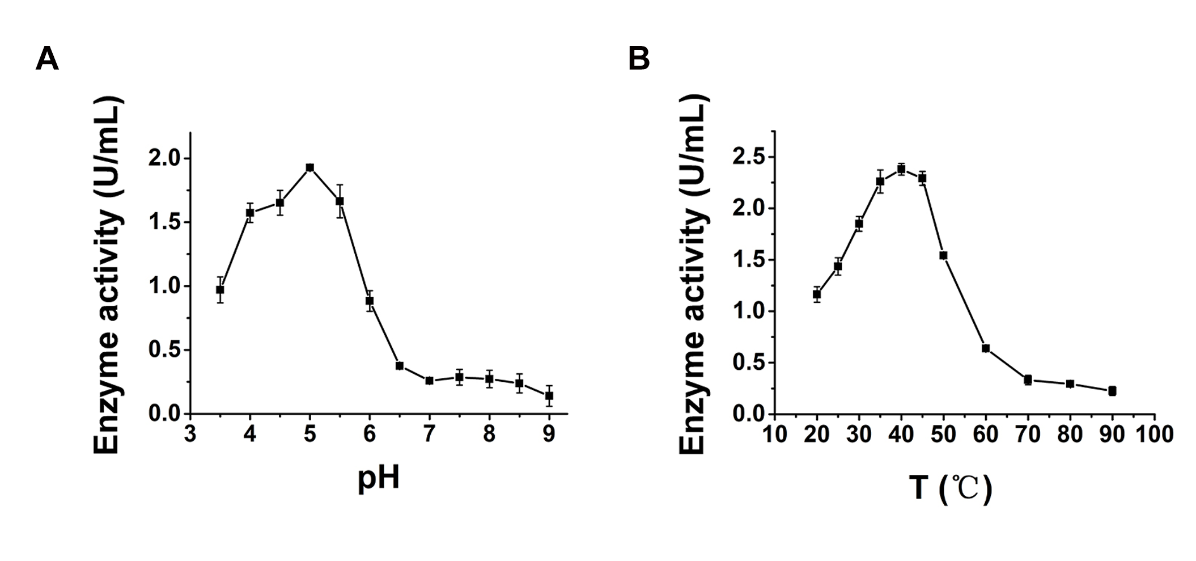


**Figure S1** Characterization of DexA70. (**A)**. The catalytic activity of DexA70 at different pH. **(B)**. The catalytic activity of DexA70 at different temperature. One unit of dextranase activity (U/mL) was defined as the amount of enzyme that degrades 1% dextran 40,000 to produce a concentration of reducing sugars equivalent to 10 micromoles of glucose min^−1^ under assay conditions. Experiments were performed at least in triplicate separately. Means ± SD are shown.
